# Isolation and Characterization of an Unknown Process-Related Impurity in Furosemide and Validation of a New HPLC Method

**DOI:** 10.3390/molecules28052415

**Published:** 2023-03-06

**Authors:** Ao Xu, Yunlin Xue, Yuyu Zeng, Jing Li, Huiling Zhou, Zhen Wang, Yin Chen, Hui Chen, Jian Jin, Tao Zhuang

**Affiliations:** 1 Jiangsu Key Laboratory of Marine Biological Resources and Environment, Jiangsu Key Laboratory of Marine Pharmaceutical Compound Screening, School of Pharmacy, Jiangsu Ocean University, Lianyungang 222005, China; 2 Co-Innovation Center of Jiangsu Marine Bio-Industry Technology, Jiangsu Ocean University, Lianyungang 222005, China; 3 Xuzhou Institute for Food and Drug Control, Xuzhou 221000, China; 4 Department of Biomedical Engineering, College of Life Science and Technology, Huazhong University of Science and Technology, Wuhan 430074, China

**Keywords:** furosemide, process-related impurity, characterization, Q-TOF/LC–MS and NMR, method validation

## Abstract

Furosemide is a widely used loop diuretic in the treatment of congestive heart failure and edema. During the preparation of furosemide, a new process-related impurity G in the levels ranging from 0.08% to 0.13% was detected in pilot batches by a new high performance liquid chromatography (HPLC) method. The new impurity was isolated and characterized by comprehensive analysis of FT-IR, Q-TOF/LC-MS, 1D-NMR (^1^H, ^13^C, and DEPT), and 2D-NMR (^1^H-^1^H-COSY, HSQC, and HMBC) spectroscopy data. The possible formation pathway of impurity G was also discussed in detail. Moreover, a novel HPLC method was developed and validated for the determination of impurity G and the other six known impurities registered in the European Pharmacopoeia as per ICH guidelines. The HPLC method was validated with respect to system suitability, linearity, the limit of quantitation, the limit of detection, precision, accuracy, and robustness. The characterization of impurity G and the validation of its quantitative HPLC method were reported for the first time in this paper. Finally, the toxicological properties of impurity G were predicted by the in silico webserver ProTox-II.

## 1. Introduction

**Figure 1:**
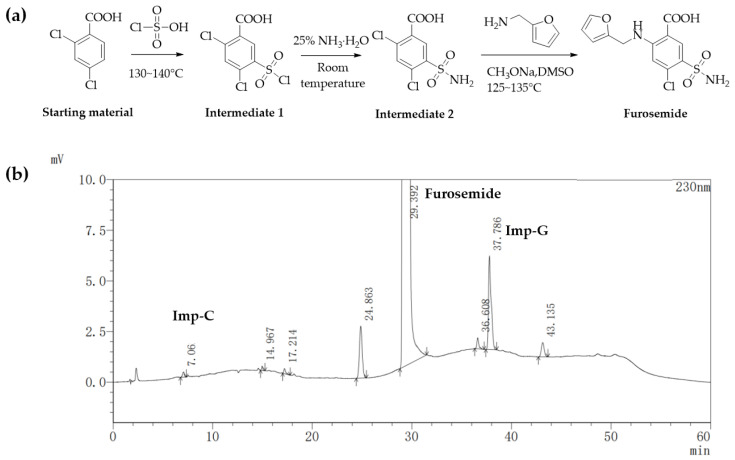
(**a**) Synthetic route of furosemide; (**b**) Representative HPLC chromatogram of furosemide with impurity G.

Furosemide, 5-(amino sulfonyl)-4-chloro-2-[(2-furanylmethyl) amino] benzoic acid (Figure 1a), is a potent loop diuretic that acts on the kidneys by inhibiting electrolyte reabsorption from the kidneys and enhancing the excretion of water from the body, which ultimately increases the water loss rate in the body [1]. It is widely used for edema secondary to various clinical conditions, such as congestive heart failure, liver failure, renal failure, and high blood pressure [2,3,4,5,6].

There are several synthetic routes of furosemide documented in the literature [7,8,9,10,11,12]. We manufactured furosemide according to the pathway given in Figure 1a for its simple, robust, and cost-effective process as well as the high yield [11,12]. In this route, the starting material 2,4-dichlorobenzoic acid underwent chlorosulfonation, ammonization, and condensation to obtain furosemide. First, 2,4-dichlorobenzoic acid was reacted with chlorosulfonic acid at 130~140 °C to obtain 2,4-dichloro-5-(chlorosulfonyl) benzoic acid (intermediate 1). The intermediate 1 then was reacted with 25% ammonia at room temperature to prepare 2,4-dichloro-5-sulfonamidobenzoic acid (intermediate 2). At last, furosemide was synthesized by the reaction of intermediate 2 with furan-2-ylmethanamine under the conditions of sodium methoxide and DMSO at 125~135 °C. During the manufacture of furosemide, an unknown process-related impurity, G, in pilot batches was detected at the levels of 0.08–0.13% by HPLC (Figure 1b).

The safety of a drug product is dependent on the toxicological properties of both the active drug substance and its impurities [13,14]. According to the requirements of ICH Q3A(R2), the reporting and identification thresholds for unknown impurities are 0.05% and 0.10% or 1.0 mg per day intake for new drug substances having a maximum daily dose ≤2 g per day, respectively [15]. Our research showed that the unknown process-related impurity G in furosemide was detected above the identification threshold and cannot be removed thoroughly during subsequent purification procedures. Therefore, the unknown impurity G needed to be identified and characterized.

**Figure 2:**
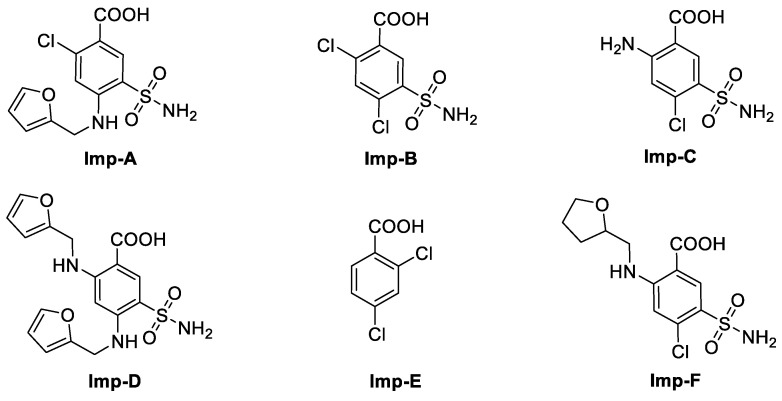
Chemical structures of impurities A–F registered in EP 10.0.

The European Pharmacopoeia (EP) 10.0 has registered six known furosemide impurities, i.e., impurities A–F (Figure 2) [16], and several articles have been reported on the stability behavior and impurities of furosemide [17,18,19,20,21,22]. However, impurity G has not been reported yet and the analytical methods in reported studies are mostly referred to EP 10.0 and the United States Pharmacopeia (USP) 43. The HPLC mobile phases described in EP for furosemide-related substances consist of potassium dihydrogen phosphate, cetrimide, and propanol. According to USP43, the HPLC mobile phases for the determination of furosemide-related substances are combinations of tetrahydrofuran, glacial acetic acid, and water (30:1:70, *v*/*v*/*v*) [23]. However, the ion-pair reagent used in EP may do irreversible harm to the chromatographic column and it is difficult to be removed from instruments. On the other hand, the use of a high proportion of tetrahydrofuran may adversely affect the health of the researchers and the instruments. The ideal analytical method should be fast, easy, robust, cost efficient, and relatively eco-friendly as it can be used not only by research laboratories but also by pharmaceutical companies [24].

As forthe continuing research efforts on impurity profiling [25,26,27], in this paper, an unknown impurity G, at a level greater than the identification threshold, was isolated from crude furosemide using column chromatography, and then we successfully resolved its structure using FT-IR, Q-TOF/LC-MS, 1D-NMR (^1^H, ^13^C, and DEPT), and 2D-NMR. A possible mechanistic pathway for the formation of impurity G was proposed in this work. Moreover, a novel HPLC method for determining unknown impurity G and six known impurities A–F in furosemide was developed and validated satisfactorily with respect to system suitability, linearity, the limit of quantitation, the limit of detection, precision, accuracy, and robustness. Finally, in silico toxicity studies were carried out using the ProTox-II web server to predict the toxicity potential of this unknown impurity G. We believe that our research will benefit the quality control of furosemide and furosemide products.

## 2. Results

### 2.1. Detection and Separation of Impurity G

Pilot batch furosemide samples were analyzed by the HPLC method as described in Section 3.2. An unknown process-related impurity G, was detected at the levels of 0.08–0.13% (Figure 1b). The retention time of furosemide was 29.392 min, and the retention times of impurity C and impurity G were 7.06 min (relative retention time: RRT 0.24) and 37.786 min (RRT 1.29), respectively. The peak area of impurity G was significantly larger than other unknown impurities, accounting for 0.13%, which was calculated by the self-calibrated method without correction factors.

### 2.2. Structural Characterization of Impurity G

The HR-ESI-MS spectral of impurity G exhibited a quasimolecular ion [M + H]^+^ at *m*/*z* 348.1002 in positive ion mode and [M − H]^−^ at *m*/*z* 346.0871 in negative ion mode (Figure S1), indicating its molecular formula to be C_16_H_17_N_3_O_4_S ([M + H]^+^, calculated 348.1013, 3.03 ppm; [M − H]^−^, calculated 346.0867, −1.15 ppm). The mass of impurity G was found to be 43.9891 Da (CO_2_) lower than that of impurity D (C_17_H_17_N_3_O_6_S). In addition, the infrared data of impurity G suggested that there were no characteristic peaks of the carbonyl group in 1650~1900 cm^−1^ and the hydroxyl group in 2500~3200 cm^−1^ (Figure S2). The above results indicated that the carboxyl group of impurity D was lost during the formation of impurity G, and it was well supported by the 1D-NMR and 2D-NMR spectral (Figures S3–S8).

**Figure 3:**
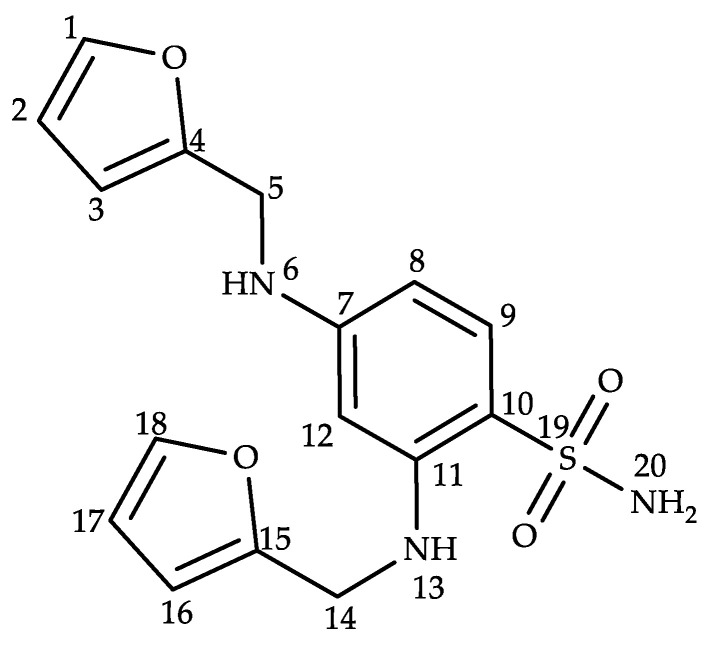
Structure of impurity G.

The ^1^H-NMR spectrum showed that there were 17 hydrogen atoms, including nine aromatic hydrogen atoms, four aliphatic hydrogen atoms, and four active hydrogen atoms, which was consistent with the molecular formula obtained from high-resolution mass spectrometry. The ^13^C-NMR and DEPT 135 spectra showed that there were 16 carbons, including five quaternary carbon atoms, two secondary carbon atoms, and nine primary or tertiary carbon atoms (Table 1). The HMBC correlations from H_6_ (δ 6.22 ppm) to C_4_ (δ 153.2 ppm), C_8_ (δ 153.2 ppm), and C_12_ (δ, 94.0 ppm) confirmed the connection between the furfurylamine group and C_7_. Likewise, another furfurylamine substituent was found to be connected with C_11_. Therefore, the structure of unknown impurity G was determined as 2,4-bis((furan-2-ylmethyl)amino)benzene sulfonamide (Figure 3).

### 2.3. Formation Pathway and Controlling of Impurity G

**Figure 4:**
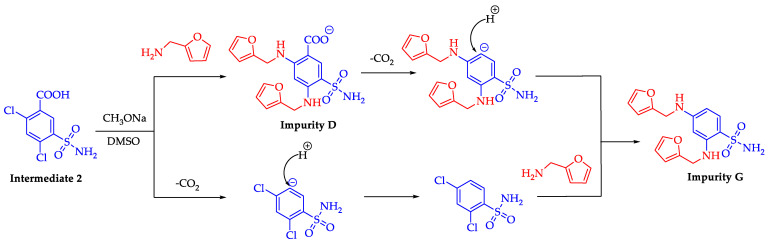
Plausible mechanism for the formation of impurity G.

During the preparation of drugs, process-related impurities might be formed due to side reactions. Based on the synthetic route employed for the preparation of furosemide, the proposed formation pathway of impurity G was shown in Figure 4. There were two possible pathways to form impurity G. First, the C-Cl bonds of 2,4-dichloro-5-sulfonamidobenzoic acid (intermediate 2) were activated due to the existence of an electron-withdrawing group (sulfonamido group) in the benzene ring. In the presence of excess furylamine, impurity D was obtained in a disubstitution reaction, and then impurity D was decarboxylated at a high temperature (125 °C ≤ T ≤ 135 °C) to give impurity G [28,29]. In another pathway, intermediate 2 was decarboxylated first, then underwent a disubstitution reaction to give impurity G.

According to the HPLC results, the content of impurity G in the furosemide samples showed a significant positive correlation with the reaction time. However, the reaction time did not obviously affect the yield of furosemide when the reaction took more than 6 h. Thus, the reaction time was set at 6 h to reduce the formation of impurity G. In the post-treatment process of the reaction solution, impurity G was partly removed with tetrahydrofuran under the condition of pH = 13–14. In addition, during the later stages of refinement, furosemide was dissolved in a saturated sodium bicarbonate aqueous solution at 65 °C, while impurity G was insoluble in a sodium bicarbonate aqueous solution so that it could be removed. Finally, the levels of impurity G were reduced to less than 0.05% with the aid of activated charcoal and polytetrafluoroethylene membrane filter (50 μm and 25 μm) for refinement.

### 2.4. Optimization of the HPLC-UV Method

Based on the chemical structure of furosemide and impurity A–G, reverse-phase liquid chromatography was suitable for the analysis of the compounds. In the preliminary experiments, different types of HPLC columns, such as Shimdzu GL Inertsustain C_18_ (150 × 4.6 mm, 5 μm) column, Waters XBridge ShieldRP C_18_ (150 × 4.6 mm, 5 μm) column, Agilent Eclipse XDB C_18_ (250 × 4.6 mm, 5 μm) column, and Agilent Eclipse XDB C_18_ (150 × 4.6 mm, 5 μm) column were tested to analyzed furosemide and its related substances (Figure S9). The best resolution was obtained using an Agilent Eclipse XDB C_18_ (150 × 4.6 mm, 5 μm) column, which was used for further optimization of the method.

**Figure 5:**
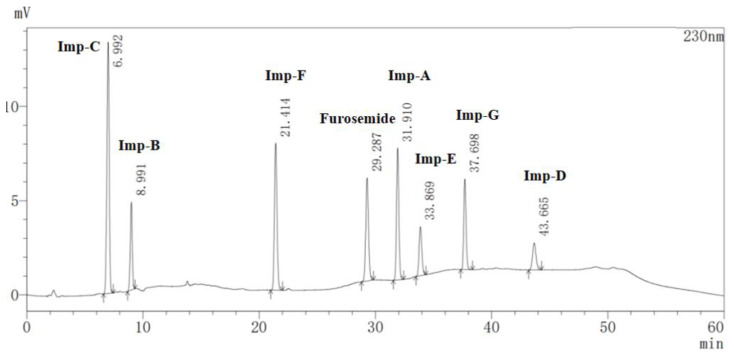
HPLC chromatogram of the specificity solution. The retention times for furosemide and impurity A–G: furosemide, 29.287 min; impurity C, 6.992 min; impurity B, 8.991 min; impurity F, 21.414 min; impurity A, 31.910 min; impurity E, 33.869 min; impurity G, 37.698 min; impurity D, 43.665 min.

The quantification of furosemide-related substances was set at 230 nm as both furosemide and impurity A–G showed strong UV absorption at 230 nm. The mixture of 0.01 mol/L KH_2_PO_4_ buffer solution and methanol was used as the mobile phase. To optimize the pH of the KH_2_PO_4_ buffer solution and gradient program of mobile phases, retention time, theoretical plates, symmetry factor, and resolution were evaluated. The optimal chromatographic behaviors were obtained when the pH of the KH_2_PO_4_ buffer solution was 3.0. Higher pH led to a loss in resolution while lower pH might result in serious harm to the chromatography column. In the meantime, the contribution of phosphoric acid to improve the baseline fluctuation was found much better than acetic acid. As the polarities of impurity B and impurity C were strong and similar, the ratio of methanol in the initial gradient elution condition and the rate of changes in the mobile gradient should be low. Impurity D was eluted out when mobile phase B was a mixture of 0.01 mol/L KH_2_PO_4_ buffer solution (pH = 3.0.) and methanol (50/50, *v*/*v*). Ultimately, good separation of impurities A–G was achieved with sharp peaks and good selectivity with mobile phase A (buffer solution and methanol (90/10, *v*/*v*)) and mobile phase B (buffer solution and methanol (50/50, *v/v*)) under the gradient conditions, time (min)/% B: 0/10, 10/63, 20/63, 30/100, 45/100, 55/10, and 60/10. The column temperature was maintained at 35 °C and the flow rate was 0.8 mL/min with a PDA detector set at 230 nm. A typical chromatogram showing the separation of the furosemide-related substances was given in Figure 5 and optimized conditions were described in Section 3.2.

### 2.5. HPLC Method Validation

The developed HPLC method was validated according to the ICH Q2(R1) guideline and established by spiking impurities into furosemide [30]. A series of validation projects were conducted to ensure the specificity, accuracy, and precision of the method, and the details are given below.

#### 2.5.1. Specificity

The specificity of the developed method was evaluated by injecting a blank solution and a specificity solution. There were no interfering coeluting peaks observed in the blank solution. As shown in Figure 5 and Table 2, furosemide and impurity A–G were found completely separated from each other, and the minimum resolution between them was 4.890. Also, the peak purity of furosemide satisfied the criteria of the PDA detector.

#### 2.5.2. Limits of Detection (LODs) and Limits of Quantitation (LOQs)

The LODs and LOQs for furosemide and impurity A–G were estimated at a signal-to-noise ratio (S/N) of 3:1 and 10:1, respectively. As shown in Table 3, the LODs of furosemide and impurity A–G were 0.012, 0.020, 0.019, 0.005, 0.098, 0.096, 0.019, and 0.020 µg/mL respectively. The LOQs of furosemide and impurity A–G were 0.061, 0.099, 0.097, 0.024, 0.488, 0.222, 0.097, and 0.059 µg/mL respectively. RSD values of peak areas for five replicate injections at LOQ concentration were found below 2.0% and the LOQs for impurity A–G were <0.05% (reporting threshold) and met the validation criterion.

#### 2.5.3. Linearity

Seven different concentrations of standard solutions, which ranged from LOQ to 200% of the normal concentration for furosemide (i.e., 0.10%) and impurity A–G (0.15%), were used to evaluate linearity. The peak area versus concentration data was analyzed with least squares linear regression. Correlation coefficients (r) of furosemide and impurity A–G were found ≥0.9999, indicating the good linearity of the method (Table 4).

#### 2.5.4. Accuracy

The accuracy was evaluated by spiked solutions at three different concentration levels (50%, 100%, and 150% of the normal concentration) and the results were presented in Table 5. The accuracy data of impurity A–G at each level were achieved within the limit range of 90–110% and the RSD of accuracy was found to be <3%.

#### 2.5.5. Repeatability and Intermediate Precision

Repeatability was performed by injecting a standard solution and six individual spiked solutions, while the same procedure was applied for the intermediate precision on a different day by a different analyst using a different batch column and a different instrument in the same laboratory. RSD values of the content in a spiked sample solution for repeatability and intermediate precision studies did not exceed 5%, indicating good precision (Table 5).

#### 2.5.6. Robustness

Robustness studies were performed by altering the existing chromatographic conditions such as column oven temperature (±3 °C), flow rate (±0.1 mL/min), pH of buffer solution (±0.1), and mobile phase composition (±2% of gradient composition). It was evaluated by relative retention time, theoretical plates, symmetry factor, and resolution of samples in a system suitability solution. Also, the content calculated by the external standard method in a spiked sample solution was compared and summarized in Table S1. Compared to the normal condition, the difference in the content of impurity A–G of altered conditions was less than 0.01%. Moreover, minor changes in the experimental parameters showed no effect on the method’s performance as gauged by theoretical plates, symmetry factor, and resolution, and the maximum difference in RRTs of impurity A–G was 0.1 (Tables S2–S5). The results revealed that the method was unaffected upon applying minor variations to the chromatographic conditions.

### 2.6. Prediction of Toxicity of Impurity G by ProTox-II Platform

The ProTox-II platform was widely used to evaluate the potential toxicity of impurities or metabolites during drug development [31,32]. According to the data shown in Table S6, furosemide, and impurity G exhibited no or low potential acute toxicity, hepatotoxicity, cytotoxicity, carcinogenicity, mutagenicity, and immunotoxicity. Additionally, furosemide and impurity G were predicted inactive for 12 different toxicological pathways.

## 3. Materials and Methods

### 3.1. Materials and Chemicals

Pilot (batch No 2103001) and crude furosemide substances were prepared by Beijing Jingfeng Pharmaceutical Group Co., Ltd. (Zibo, Shandong, China). Reference standard furosemide (≥99.3%) was purchased from the China National Institute for Food and Drug Control (Beijing, China). Furosemide impurities A, C, and D were produced by Shandong Bolode Bio-Technology Co., Ltd. (Jinan, Shandong, China). Furosemide impurities B, E, and F were produced from Cato Research Chemicals Inc. (Eugene, OR, USA), and 2,4-dichloro-5-sulfamoylbenzoicacid was produced by Hubei Xinkang Pharmaceutical Chemical Co., Ltd. (Tianmen, Hubei, China). HPLC-grade solvents (acetonitrile, phosphoric acid, and methanol) were purchased from Thermo Fisher Scientific Inc. (Waltham, MA, USA). Distilled water was purchased from Wahaha Group Co., Ltd. (Hangzhou, Zhejiang, China). All other AR-grade reagents were purchased from Shanghai Aladdin Biochemical Technology Co., Ltd. (Shanghai, China).

### 3.2. Analytical HPLC

All samples were analyzed using a Shimadzu LC-20AD HPLC system equipped with an SPD-M20A detector and LC solution software (Shimadzu Corporation, Kyoto, Japan). An Agilent Eclipse XDB C18 column (150 × 4.6 mm, 5 µm, Agilent Technologies, Santa Clara, CA, USA) was used for the analysis, and the column temperature was set at 35 °C. Furosemide samples were dissolved in a solvent mixture (acetonitrile/water/glacial acetic acid = 500:500:0.1, *v*/*v*/*v*). The buffer solution was prepared by dissolving 1.36 g of KH_2_PO_4_ in 1000 mL of water and the pH was adjusted to 3.0 with phosphoric acid. The mixture of buffer solution and methanol (90/10, *v*/*v*) was used as mobile phase A, and mobile phase B was a mixture of buffer solution and methanol (50/50, *v*/*v*). The LC gradient program was set as follows: time (min)/% B: 0/10, 10/63, 20/63, 30/100, 45/100, 55/10, and 60/10. The injection volume was 10 μL. The flow rate was 0.8 mL/min with a detection wavelength of 230 nm.

### 3.3. HPLC-MS Analysis

High-resolution MS data was acquired on a quadrupole time-of-flight mass spectrometer (Q-TOF LC/MS G6230B, Agilent Technologies, Santa Clara, CA, USA). The protonated and ionized mass spectra were obtained using an electrospray ionization (ESI) source. The mass parameters were set in line as follows: capillary voltage, 4000 V or 3500 V; fragmentor voltage, 135 V; drying gas (N_2_) flow rate, 7.5 L/min; sheath gas (N_2_) flow rate, 10.0 L/min; drying gas temperature, 350 °C; sheath gas temperature, 300 °C; and nebulizer gas pressure, 35 psi. Mass spectra were collected in the range of m/z 100–2000 for MS. The mobile phase was 0.01% formic acid (A) and methanol (B) mixed at a ratio of 20:80 and isocratic elution was employed. The flow rate was set at 1.0 mL/min and the injection volume was 20 μL.

### 3.4. Nuclear Magnetic Resonance Spectroscopy (NMR)

The 1D NMR (^1^H, ^13^C, and DEPT) and 2D NMR (^1^H-^1^H-COSY, HSQC, and HMBC) experiments were performed on a Bruker Advance II 500 NMR instrument (Bruker, Karlsruhe, Germany) using tetramethylsilane (TMS) as an internal standard and DMSO-d_6_ as solvent. Coupling constants (J) were given in units of Hz. 2D NMR experiments including ^1^H-^1^H-COSY, HSQC, and HMBC were carried out to complete the assignments of individual peaks.

### 3.5. Fourier Transform Infrared Spectroscopy (FT-IR)

FT-IR data of furosemide and impurity G was performed on a Thermo Scientific Nicolet iS5 FT-IR spectrophotometer (Thermo Fisher Scientific, Waltham, MA, USA) and recorded in the solid state as KBr dispersion. OMNIC software was used to perform data processing and analysis. Samples were recorded over a spectral range of 4000−400 cm^−1^ and at a resolution of 2 cm^−1^.

### 3.6. Isolation of Impurity G

Dimethyl sulfoxide (DMSO) (90 g) and intermediate 2 (30 g, 111.5 mmol) were added to a 250 mL reaction flask. The reaction mixture was stirred while sodium methoxide (6.6 g, 122.67 mmol) and furan-2-ylmethanamine (32.45 g, 334.5 mmol) were added. The solution was maintained at 135 °C for 7 h and cooled to room temperature, poured into 450 g of water, then basified with a sodium hydroxide solution to pH 13 and extracted twice with tetrahydrofuran. The combined organic layer was evaporated to dryness solid. The solid was applied to a silica gel column chromatography (2.5 × 50 cm, 100–200 mesh) and eluted with dichloromethane-methanol (20:1 [*v*/*v*]) and purified by recrystallization with dichloromethane to yield impurity G with 99.5% HPLC purity.

### 3.7. Preparation of Solutions

#### 3.7.1. Preparation of Specificity Solution and Standard Solution

The blank solution and sample solvent were acetonitrile/water/glacial acetic acid = 500:500:0.1 (*v*/*v*/*v*). The stock solution was prepared by weighing impurity A–G and the furosemide reference substance appropriately and diluted with diluent (Section 3.2. Analytical HPLC) to the concentration of 150 µg/mL and 100 µg/mL, respectively. Then each stock solution was transferred into the same volumetric flask and prepared with diluent to obtain a solution with 1.5 µg/mL impurity A–G and 1.0 µg/mL furosemide. The obtained solution was used both as a specificity solution and a standard solution.

#### 3.7.2. Preparation of Sample Solution

The sample solution was prepared by weighing 10.0 mg of furosemide substance into a 10 mL volumetric flask and diluted to volume.

#### 3.7.3. Preparation of Spiked Solution for Method Validation

The spiked solution was prepared by adding 1.0 mL of the stock solution of each impurity to a 10 mL volumetric flask with a 10.0 mg furosemide sample, then diluted to volume to obtain a spiked sample solution containing each impurity at the 0.15% level.

### 3.8. Toxicity Prediction of Furosemide and Impurity G

The prediction was conducted by in silico methods using the ProTox-II platform [33,34]. Usually, toxicities are investigated at the expense of lots of time and the lives of animals. Comparatively, the ProTox-II platform provided a fast and inexpensive method to predict the toxicity of compounds. The only requirement to carry out the prediction was the two-dimensional structure of the molecule. Oral toxicity, organ toxicity, and four toxicity endpoints were evaluated for the toxicity prediction. Acute toxicity was analyzed with the two-dimensional similarity to compounds with known toxic effects and the existence of toxic fragments. Hepatotoxicity was assessed through the synthetic minority over-sampling technique (SMOTE) sampling and random forest classifier. Cytotoxicity, carcinogenicity, and mutagenicity were evaluated using machine learning with over sampling. The prediction of immunotoxicity was based on a multinomial naïve bayes learning algorithm. The toxicology in the 21st century (Tox21) data consisted of 12 pathways based on cellular assays, under two types of pathways. It should be noted that in silico methods only evaluate the potential toxicity of impurities and unexpected toxic effects cannot be ruled out.

## 4. Conclusions

A novel process-related impurity, G, that cannot be removed thoroughly was identified in pilot batches of furosemide substances and isolated through column chromatography. Structural elucidations of impurity G were conducted using FT-IR, Q-TOF/LC–MS, 1D NMR (^1^H, ^13^C, and DEPT), and 2D NMR (^1^H-^1^H-COSY, HSQC, and HMBC), and confirmed as 2,4-bis((furan-2-ylmethyl)amino)benzene sulfonamide. Possible mechanisms for the formation of impurity G were proposed and the key points for its control were elaborated. The in silico toxicity prediction of furosemide and impurity G was conducted and compared, and impurity G showed a low risk of toxicity. Meanwhile, a new HPLC method was developed and validated for the determination of six known impurities and impurity G, which provided a useful reference for quality control in the manufacture of furosemide.

## Figures and Tables

**Table 1:** ^1^H,^13^C-NMR, and 2D-NMR data for impurity G.

Position	δ_H_ (ppm)	δ_C_ (ppm)	DEPT	^1^H-^1^H COSY	HMBC
1	7.58 (d, *J* = 1.0 Hz, 1H)	142.5	CH	H_2_(^3^*J*_HH_)	C_3_(^3^*J*_CH_), C_4_(^3^*J*_CH_)
2	6.38 (dd, *J* = 3.0, 1.9 Hz, 1H)	110.8	CH	H_1_(^3^*J*_HH_), H_3_(^3^J_HH_)	C_4_(^3^*J*_CH_), C_3_(^2^*J*_CH_)
3	6.28 (d, *J* = 3.1 Hz, 1H)	107.6	CH	H_2_(^3^*J*_HH_)	C_1_(^3^*J*_CH_), C_2_(^2^*J*_CH_), C_4_(^2^*J*_CH_)
4	–	153.2	–	–	–
5	4.27 (d, *J* = 4.6 Hz, 2H)	39.8	CH_2_	–	C_3_(^3^*J*_CH_), C_4_(^2^*J*_CH_)
6	6.22 (t, *J* = 5.4 Hz, 1H)	–	–	–	C_12_(^3^*J*_CH_), C_8_(^3^*J*_CH_), C_4_(^3^*J*_CH_), C_5_(^2^*J*_CH_)
7	–	113.8	–	–	–
8	6.02 (d, *J* = 3.0 Hz, 1H)	101.2	CH	H_9_(^3^*J*_HH_)	C_4_(^5^*J*_CH_), C_12_(^3^*J*_CH_), C_7_(^2^*J*_CH_), C_9_(^2^*J*_CH_)
9	7.35 (d, *J* = 9.2 Hz, 1H)	130.4	CH	H_8_(^3^*J*_HH_)	C_15_(^6^*J*_CH_), C_12_(^4^*J*_CH_), C_11_(^3^*J*_CH_), C_10_(^2^*J*_CH_)
10	–	146.4	–	–	–
11	–	113.9	–	–	–
12	6.01 (d, *J* = 2.2 Hz, 1H)	94.0	–	–	C_15_(^5^*J*_CH_), C_9_(^4^*J*_CH_), C_8_(^3^*J*_CH_), C_7_(^2^*J*_CH_)
13	6.62 (t, *J* = 5.2 Hz, 1H)	–	CH	–	C_11_(^3^*J*_CH_), C_15_(^3^*J*_CH_), C_14_(^2^*J*_CH_), C_12_(^2^*J*_CH_)
14	4.34 (d, *J* = 4.6 Hz, 2H)	40.2	CH_2_	–	C_10_(^4^*J*_CH_), C_16_(^3^*J*_CH_), C_15_(^2^*J*_CH_)
15	–	152.9	–	–	–
16	6.34 (d, *J* = 3.1 Hz, 1H)	107.5	CH	H_17_(^3^*J*_HH_)	C_18_(^3^*J*_CH_), C_15_(^2^*J*_CH_), C_17_(^2^*J*_CH_)
17	6.43 (dd, *J* = 3.0, 1.9 Hz, 1H)	110.9	CH	H_18_(^3^*J*_HH_), H_16_(^3^*J*_HH_)	C_15_(^3^*J*_CH_), C_16_(^2^*J*_CH_), C_18_(^2^*J*_CH_)
18	7.60 (d, *J* = 0.9 Hz, 1H)	142.7	CH	H_17_(^3^*J*_HH_)	C_15_(^3^*J*_CH_), C_16_(^3^*J*_CH_)
20	6.92 (s, 2H)	–	–	–	–

**Table 2:** Results for specificity.

Compound	Relative Retention Time (RRT)	Resolution	Theoretical Plates	Symmetry Factor
Furosemide	1.00	6.517	71,898	1.087
Imp-A	1.09	4.890	120,398	1.097
Imp-B	0.31	34.377	12,953	0.952
Imp-C	0.24	–	5670	0.931
Imp-D	1.49	5.815	100,376	1.089
Imp-E	1.16	9.637	97,672	1.129
Imp-F	0.73	18.664	44,339	1.097
Imp-G	1.29	13.078	174,284	1.119

**Table 3:** LODs and LOQs data of furosemide and impurity A–G.

Compound	LOD			LOQ			
	µg/mL	%	S/N	µg/mL	%	S/N	RSD (%)
Furosemide	0.012	0.0012	4.11	0.061	0.0061	14.27	0.76
Imp-A	0.020	0.0020	3.59	0.099	0.0099	17.82	0.78
Imp-B	0.019	0.0019	2.70	0.097	0.0097	13.02	1.48
Imp-C	0.005	0.0005	2.63	0.024	0.0024	11.82	0.35
Imp-D	0.098	0.0098	3.07	0.488	0.0488	16.18	1.04
Imp-E	0.096	0.0044	3.27	0.222	0.0222	16.37	0.65
Imp-F	0.019	0.0019	4.14	0.097	0.0097	19.46	0.58
Imp-G	0.020	0.0020	4.33	0.059	0.0059	13.04	1.61

**Table 4:** Results for linearity and range studies.

Compound	Concentration (µg/mL)	Correlation Coefficient (r)	Regression Equation
Furosemide	0.100–1.998	0.9999	y = 59,180 x + 6139
Imp-A	0.146–2.917	1.0000	y = 66,308 x − 59
Imp-B	0.150–2.993	1.0000	y = 39,245 x + 47
Imp-C	0.146–2.911	1.0000	y = 130,336 x + 325
Imp-D	0.148–2.960	1.0000	y = 20,195 x − 124
Imp-E	0.149–2.985	1.0000	y = 30,029 x + 1
Imp-F	0.145–2.904	1.0000	y = 82,955 x + 332
Imp-G	0.153–3.057	1.0000	y = 56,489 x + 236

**Table 5:** Results for accuracy, repeatability, and intermediate precision studies of impurity A–G.

Compound	Accuracy				Repeatability	Intermediate Precision
	50%	100%	150%	RSD% (*n* = 9)	RSD% (*n* = 6)	RSD% (*n* = 6)
Imp-A	101.4	101.2	101.7	0.30	2.82	2.59
Imp-B	102.2	102.1	102.0	0.47	2.88	2.70
Imp-C	102.1	102.0	102.0	0.33	2.88	1.42
Imp-D	105.5	102.8	103.6	1.50	2.86	3.94
Imp-E	101.6	101.3	102.1	0.52	2.82	1.93
Imp-F	102.3	102.0	102.0	0.33	2.90	4.26
Imp-G	103.5	104.8	104.1	2.27	2.48	2.43

## Data Availability

Not applicable.

## Supplementary Materials

The following supporting information can be downloaded at: www.mdpi.com/article/10.3390/molecules28052415/s1, The HR-ESI-MS, 1D NMR (^1^H, ^13^C, and DEPT), and 2D NMR (^1^H-^1^H-COSY, HSQC, and HMBC) spectra (Figures S1–S8); The HPLC chromatograms of different types of HPLC columns were presented (Figure S9); Results for robustness study of newly developed HPLC method (Tables S1–S5) and in silico toxicity prediction of furosemide and impurity G (Table S6) can be found in Supplementary Materials.
