# Supplementary material for: Isolation and Characterization of an Unknown Process-Related Impurity in Furosemide and Validation of a New HPLC Method
Source: Molecules. 2023 Mar 6;28(5):2415. doi: 10.3390/molecules28052415 (PMC10005432; doi:10.3390/molecules28052415)
Supplement: Supplementary file 1 [file molecules-28-02415-s001.zip › molecules-2251568-supplementary.pdf]

Isolation and Characterization of an Unknown Process-Related Impurity in Furosemide and Validation of a New HPLC Method

Ao Xu [1,2], Yunlin Xue [1,2], Yuyu Zeng [1,2], Jing Li [1,2], Huiling Zhou [1,2], Zhen Wang [1,2], Yin Chen [1,2], Hui Chen [3], Jian Jin [1,2,*] and Tao Zhuang [1,2,4,*]

[1] Jiangsu Key Laboratory of Marine Biological Resources and Environment, Jiangsu Key Laboratory of Marine Pharmaceutical Compound Screening, School of Pharmacy, Jiangsu Ocean University, Lianyungang 222005, China;

[2] Co-Innovation Center of Jiangsu Marine Bio-industry Technology, Jiangsu Ocean University, Lianyungang 222005, China

[3] Xuzhou Institute for Food and Drug Control, Xuzhou 221000, China;

[4] Department of Biomedical Engineering, College of Life Science and Technology, Huazhong University of Science and Technology, Wuhan 430074, China.

[*] Correspondence: 2019000016@jou.edu.cn (J.J.); zhuang_tao@hotmail.com (T.Z.);

Supporting Information

Table of contents

Figure S1 The HR-ESI-MS spectrum of impurity G P3

Figure S2 The IR spectrogram of furosemide and impurity G P4

Figure S3 The ^{1}H NMR spectrum of impurity G P5

Figure S4 The ^{13}C NMR spectrum of impurity G P5

Figure S5 The DEPT spectrum of impurity G P6

Figure S6 The ^{1}H-^{1}H COSY spectrum of impurity G P6

Figure S7 The HSQC spectrum of impurity G P7

Figure S8 The HMBC spectrum of impurity G P7

Figure S9 The HPLC chromatograms of different types of HPLC columns P8

Table S1 Results for robustness study of impurity A–G P9

Table S2 Results for robustness study (flow rate, ± 0.1 mL/min) P10

Table S3 Results for robustness study (column oven temperature, ± 3 °C) P11

Table S4 Results for robustness study (pH of buffer solution, ± 0.1) P12

Table S5 Results for robustness study (mobile phase composition, ± 2%) P13

Table S6 In silico toxicity prediction of furosemide and impurity G P14

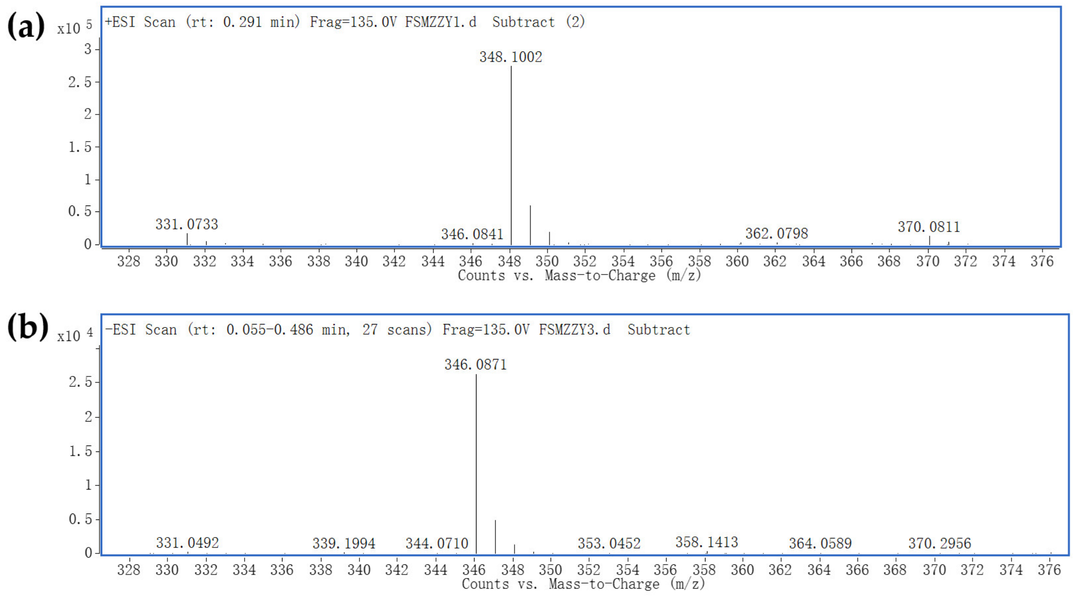

Figure S1 The HR-ESI-MS spectrum of impurity G

(a) The HR-ESI-MS spectrum of impurity G in positive ion mode

(b) The HR-ESI-MS spectrum of impurity G in negative ion mode

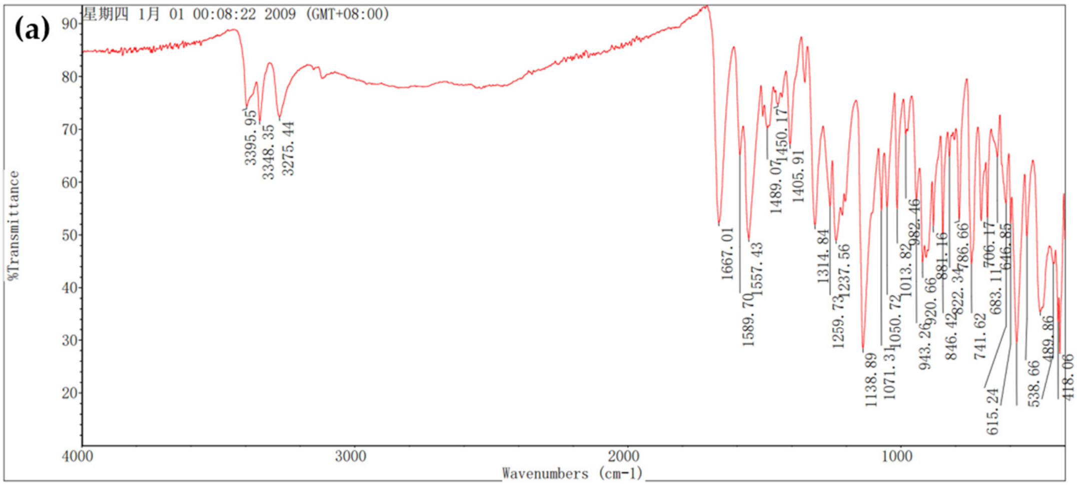

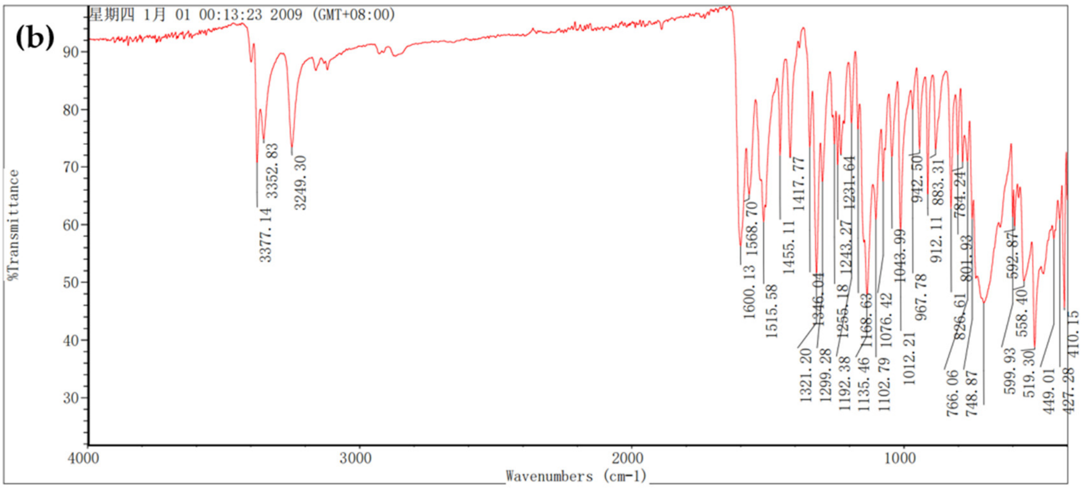

Figure S2 The IR spectra of furosemide and impurity G

(a) The IR spectrum of furosemide

(b) The IR spectrum of impurity G

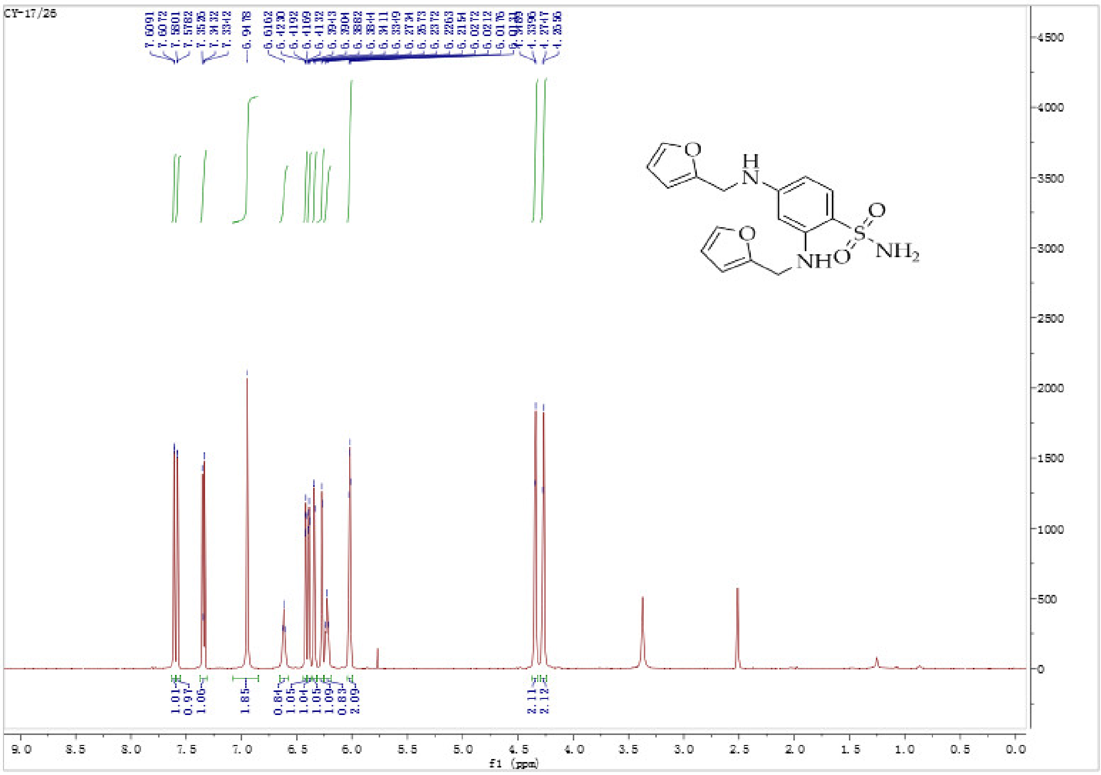

Figure S3 The ^{1}H NMR spectrum of impurity G

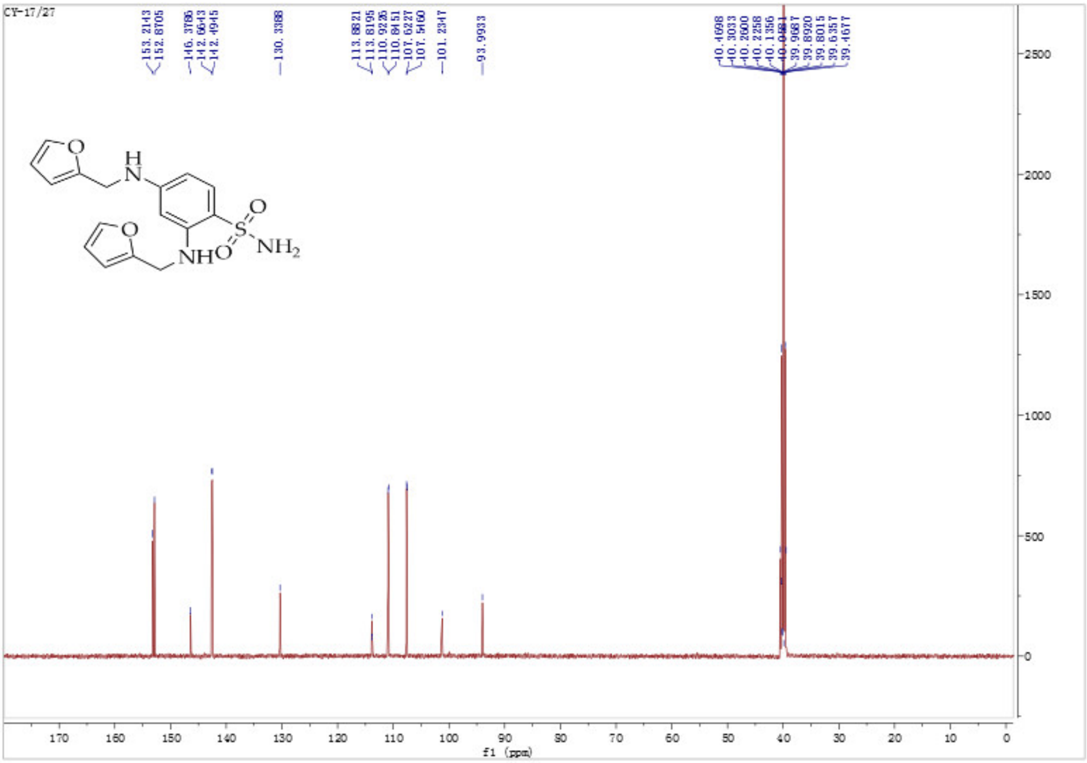

Figure S4 The ^{13}C NMR spectrum of impurity G

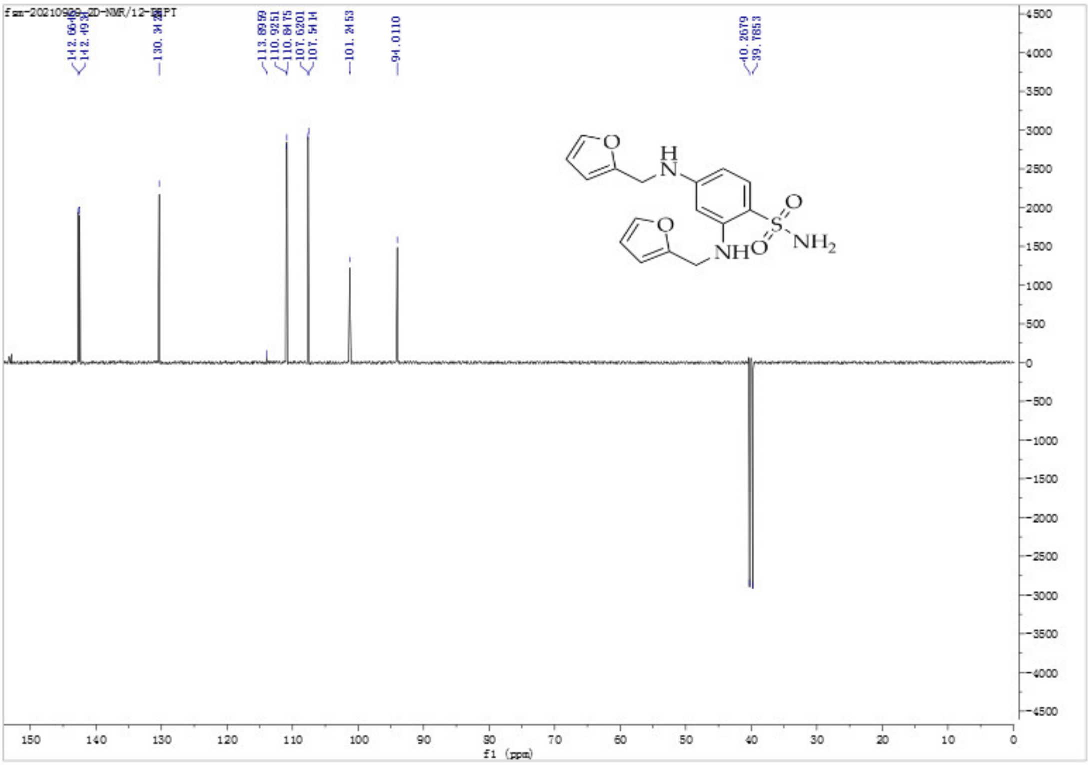

Figure S5 The DEPT spectrum of impurity G

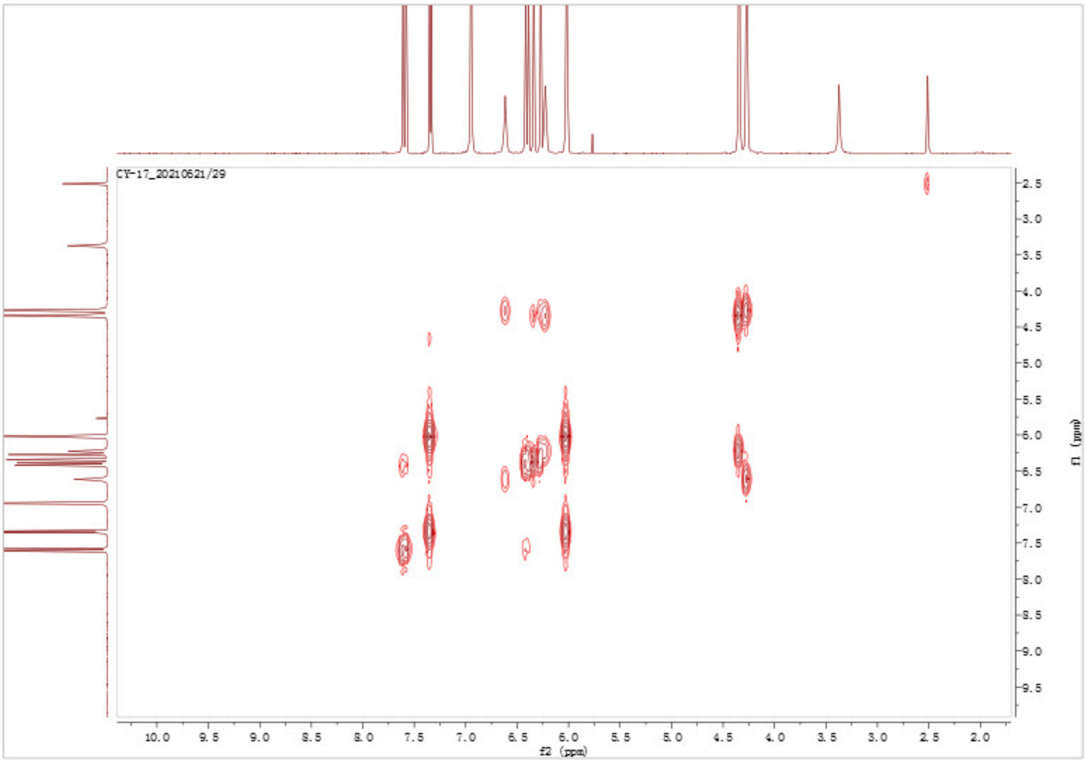

Figure S6 The ^{1}H-^{1}H COSY spectrum of impurity G

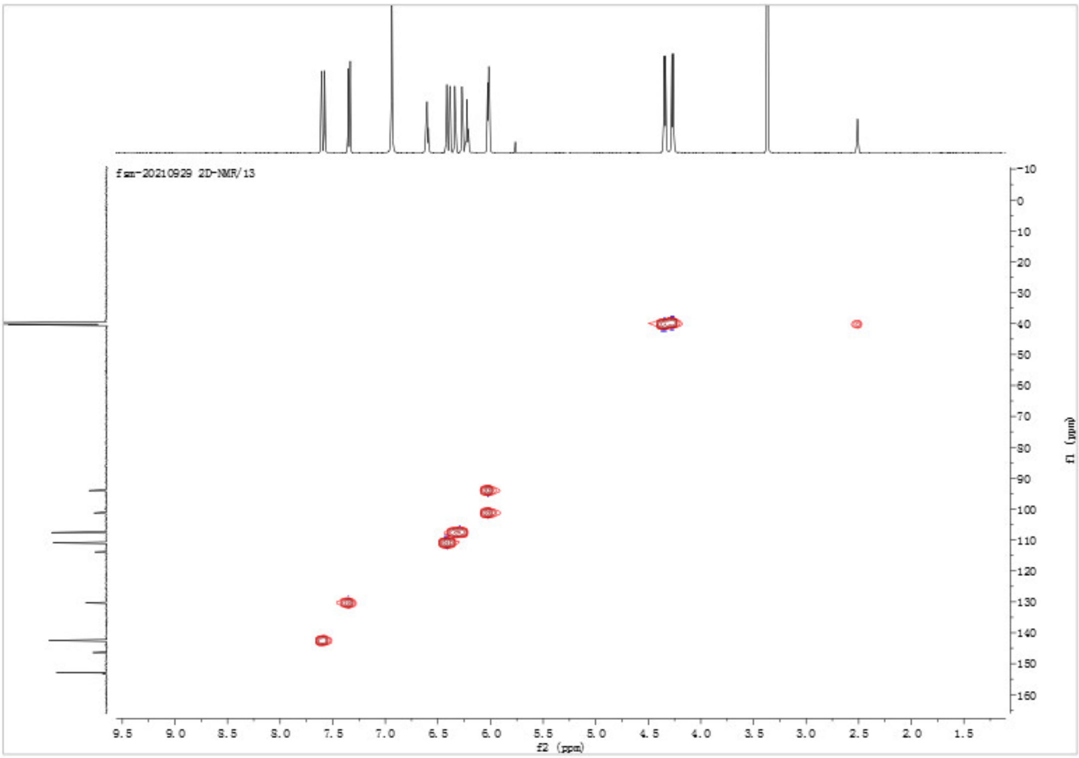

Figure S7 The HSQC spectrum of impurity G

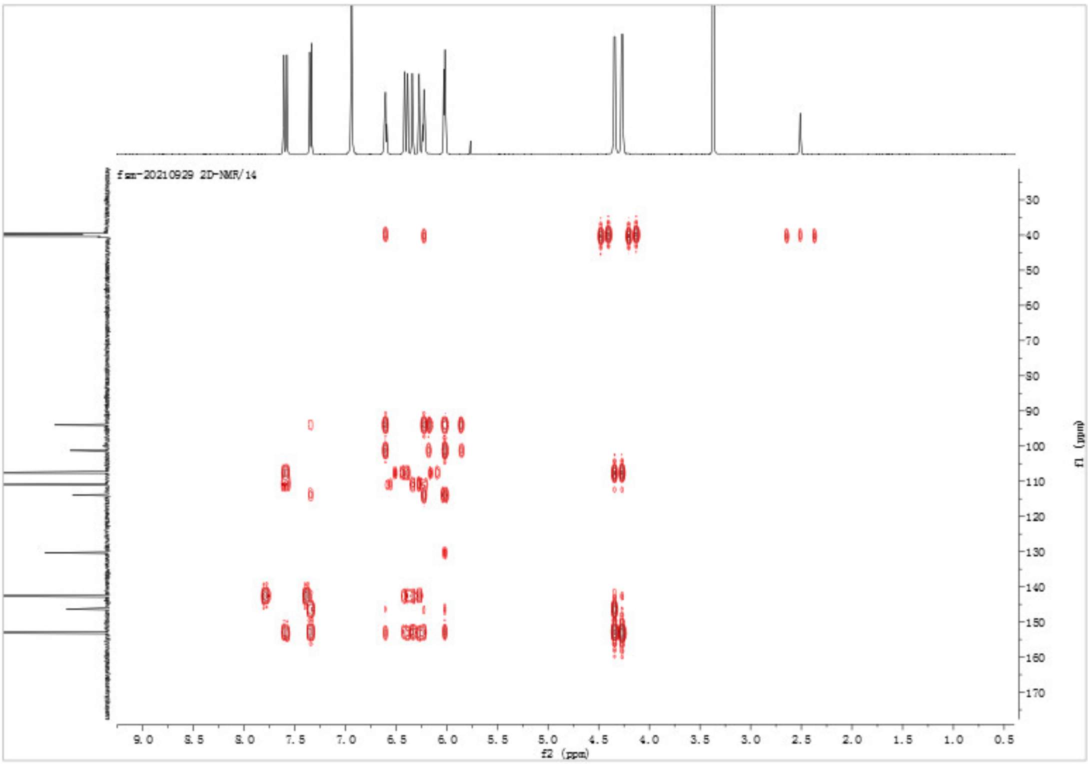

Figure S8 The HMBC spectrum of impurity G

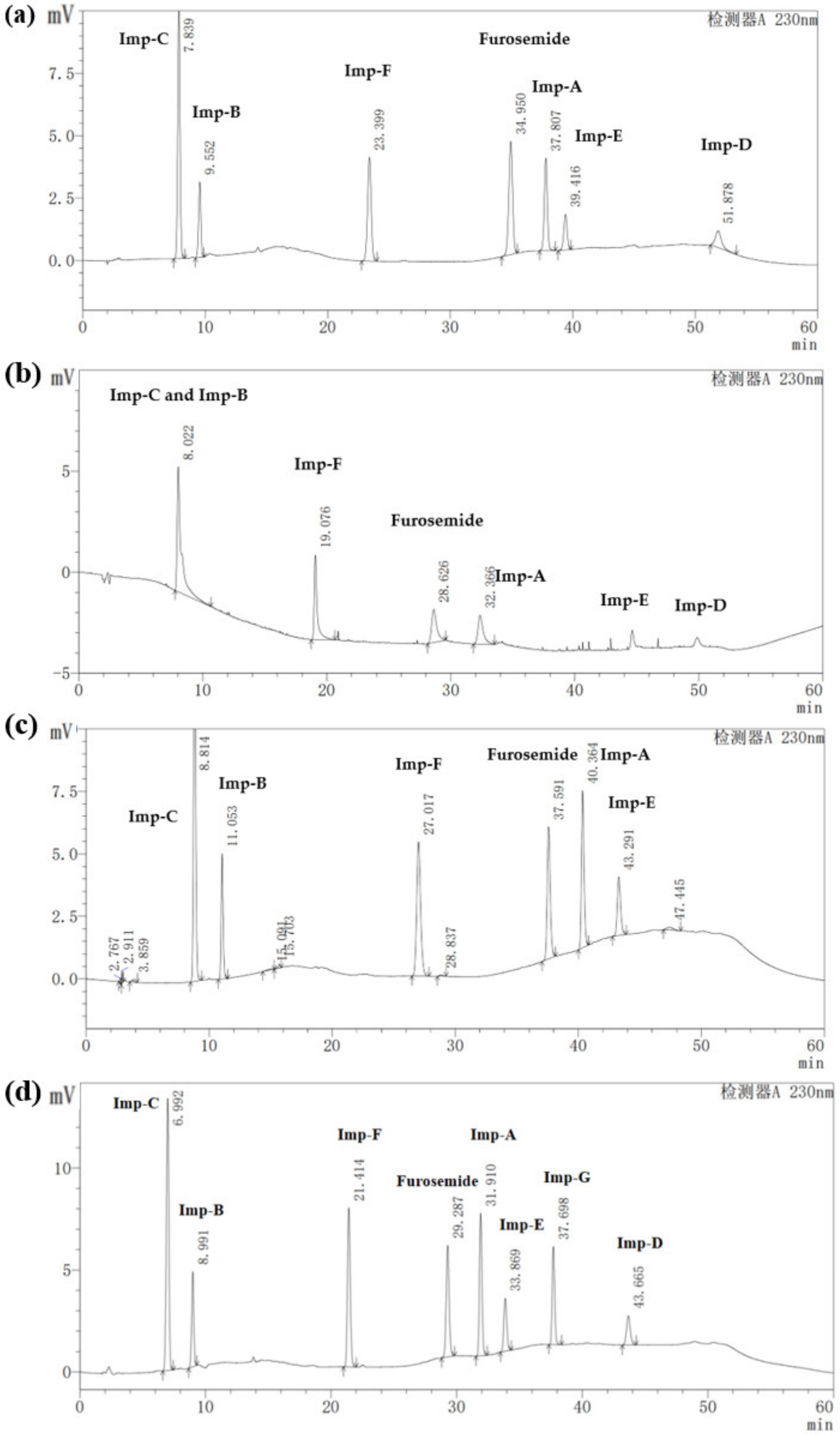

Figure S9 The HPLC chromatograms of different types of HPLC columns

(a) The HPLC chromatogram of Shimdzu GL Inertsustain C_{18} (150 × 4.6 mm, 5μm) column

(b) The HPLC chromatogram of Waters XBridge ShieldRP C_{18} (150 × 4.6 mm, 5μm) column

(c) The HPLC chromatogram of Agilent Eclipse XDB C_{18} (250 × 4.6 mm, 5μm) column

(d) The HPLC chromatogram of Agilent Eclipse XDB C_{18} (150 × 4.6 mm, 5μm) column

Table S1 Results for robustness study of impurity A–G

Parameter Altered	Variation	Content of impurity A–G (%)						
		Imp-A	Imp-B	Imp-C	Imp-D	Imp-E	Imp-F	Imp-G
Normal conditions *		0.14	0.14	0.14	0.14	0.14	0.13	0.13
Flow rate	- 0.1mL/min	0.14	0.14	0.14	0.14	0.14	0.14	0.13
	+ 0.1mL/min	0.14	0.14	0.14	0.14	0.14	0.14	0.14
Column oven Temperature	- 3 °C	0.13	0.14	0.14	0.14	0.14	0.14	0.14
	+ 3 °C	0.14	0.14	0.14	0.14	0.14	0.14	0.14
pH	- 0.1	0.14	0.15	0.14	0.14	0.14	0.13	0.13
	+ 0.1	0.14	0.15	0.14	0.14	0.14	0.13	0.13
%Organic in Mobile phase A	- 2%	0.14	0.15	0.14	0.14	0.14	0.14	0.13
	+ 2%	0.14	0.15	0.14	0.14	0.14	0.14	0.13
%Organic in Mobile phase B	- 2%	0.14	0.14	0.14	0.14	0.14	0.14	0.13
	+ 2%	0.14	0.14	0.14	0.14	0.14	0.14	0.13

* The normal conditions was described in section 3.2.

Table S2 Results for robustness study (flow rate, ± 0.1 mL/min)

Flow (mL/min)	Compound	System suitability				
		RRT (min)	RT (min)	Theoretical plates	Symmetry factor	Resolution
0.7	Imp C	0.27	8.441	7,567	0.933	-
	Imp B	0.34	10.807	17,425	0.920	6.544
	Imp F	0.75	23.997	44,604	1.035	34.033
	Furosemide	1.00	31.844	104,702	1.056	18.539
	Imp A	1.07	34.228	154,939	1.053	6.452
	Imp E	1.15	36.50	123,688	1.088	11.997
	Imp G	1.27	40.499	163,554	1.117	14.707
	Imp D	1.46	46.639	93,079	1.047	6.021
0.9	Imp C	0.24	6.700	6,843	0.941	-
	Imp B	0.31	8.652	13,913	0.935	6.478
	Imp F	0.73	20.255	45,835	1.057	34.744
	Furosemide	1.00	27.886	58,481	1.019	18.234
	Imp A	1.10	30.633	107,382	1.053	6.616
	Imp E	1.16	32.37	88,405	0.999	4.226
	Imp G	1.29	36.029	148,050	1.065	13.225
	Imp D	1.50	41.754	110,642	1.055	5.847

Table S3 Results for robustness study (column oven temperature, ± 3 °C)

Temperature (°C)	Compound	System suitability				
		RRT (min)	RT (min)	Theoretical plates	Symmetry factor	Resolution
32	Imp C	0.26	7.820	8,697	0.962	-
	Imp B	0.31	9.548	15,159	0.979	5.416
	Imp F	0.74	22.751	41,799	1.080	35.068
	Furosemide	1.00	30.659	96,195	1.098	18.918
	Imp A	1.08	33.066	143,891	1.069	6.413
	Imp E	1.13	34.62	109,152	1.092	3.972
	Imp G	1.27	38.998	128,444	1.113	13.666
	Imp D	1.48	45.351	89,852	1.044	5.483
38	Imp C	0.24	6.971	5,803	0.854	-
	Imp B	0.32	9.179	13,535	0.867	6.493
	Imp F	0.74	21.048	49,307	1.039	34.212
	Furosemide	1.00	28.561	63,138	1.034	18.019
	Imp A	1.10	31.307	112,539	1.035	6.492
	Imp E	1.17	33.35	95,478	1.011	5.177
	Imp G	1.29	36.932	133,651	1.071	14.060
	Imp D	1.49	42.511	116,857	1.038	5.839

Table S4 Results for robustness study (pH of buffer solution, ± 0.1).

pH	Compound	System suitability				
		RRT (min)	RT (min)	Theoretical plates	Symmetry factor	Resolution
2.9	Imp C	0.25	7.475	7,372	0.938	-
	Imp B	0.33	9.824	17,008	1.001	7.184
	Imp F	0.74	21.859	46,961	1.063	34.300
	Furosemide	1.00	29.688	82,180	1.034	19.159
	Imp A	1.09	32.239	133,980	1.042	6.682
	Imp E	1.17	34.65	112,882	1.025	6.300
	Imp G	1.26	37.394	125,804	1.044	15.259
	Imp D	1.46	43.475	105,508	1.018	6.150
3.1	Imp C	0.25	7.148	6,529	0.941	-
	Imp B	0.29	8.551	10,495	0.904	4.095
	Imp F	0.73	21.432	48,062	1.041	35.647
	Furosemide	1.00	29.171	71,538	1.031	18.753
	Imp A	1.09	31.695	118,656	1.029	6.358
	Imp E	1.11	32.45	83,897	1.027	1.828
	Imp G	1.27	36.908	129,621	1.037	15.840
	Imp D	1.50	43.776	101,838	1.043	6.296

Table S5 Results for robustness study (mobile phase composition, ± 2%)

Mobile phase composition		Compound	System suitability				
			RRT (min)	RT (min)	Theoretical plates	Symmetry factor	Resolution
%Organic in Mobile phase A	- 2%	Imp C	0.25	7.489	7,395	0.915	-
		Imp B	0.31	9.143	13,141	0.911	4.905
		Imp F	0.74	21.742	46,649	1.078	35.036
		Furosemide	1.00	29.474	77,334	1.068	18.711
		Imp A	1.08	31.958	126,478	1.090	6.449
		Imp E	1.13	33.25	95,851	1.145	3.304
		Imp G	1.26	37.278	118,640	1.104	14.988
		Imp D	1.47	43.412	110,129	1.057	5.694
	+ 2%	Imp C	0.24	6.706	5,063	0.897	-
		Imp B	0.29	8.350	9,574	0.886	4.664
		Imp F	0.73	20.809	48,736	1.093	34.986
		Furosemide	1.00	28.408	58,286	1.091	17.932
		Imp A	1.10	31.170	103,329	1.095	6.413
		Imp E	1.14	32.46	82,222	1.048	3.029
		Imp G	1.29	36.596	108,149	1.061	14.499
		Imp D	1.51	42.981	110,384	1.042	5.952
%Organic in Mobile phase B	- 2%	Imp C	0.26	7.430	7,082	0.937	-
		Imp B	0.32	9.056	12,802	0.927	4.817
		Imp F	0.74	21.097	49,216	1.080	34.553
		Furosemide	1.00	28.575	65,597	1.074	18.143
		Imp A	1.09	31.192	114,153	1.079	6.360
		Imp E	1.14	32.56	89,141	1.127	3.406
		Imp G	1.26	36.136	122,087	1.083	14.010
		Imp D	1.47	41.905	127,114	1.062	5.314
	+ 2%	Imp C	0.25	7.568	7,130	0.929	-
		Imp B	0.30	9.249	12,995	0.885	4.898
		Imp F	0.74	22.562	43,374	1.094	35.332
		Furosemide	1.00	30.442	90,600	1.088	18.701
		Imp A	1.08	32.816	139,811	1.077	6.335
		Imp E	1.12	33.98	101,714	1.038	2.989
		Imp G	1.20	36.665	108,728	1.050	13.469
		Imp D	1.50	45.552	91,305	1.079	22.504

Table S6 In silico toxicity prediction of furosemide and impurity G

Classification	Target	Furosemide		Impurity G	
		Prediction	probability	Prediction	probability
Oral toxicity	LD_{50} (mg/kg)	2000	–	3000	–
	Toxicity Class	4	–	5	–
Organ toxicity	Hepatotoxicity	Inactive	0.51	Inactive	0.64
Toxicity endpoints	Carcinogenicity	Inactive	0.62	Inactive	0.53
	Mutagenicity	Inactive	0.89	Inactive	0.73
	Immunotoxicity	Inactive	0.99	Inactive	0.93
	Cytotoxicity	Inactive	0.59	Inactive	0.58
Tox21-Nuclear receptor signaling pathways	Aryl hydrocarbon receptor	Inactive	0.97	Inactive	0.92
	Androgen receptor	Inactive	0.97	Inactive	0.93
	Androgen receptor ligand binding domain	Inactive	0.98	Inactive	0.94
	Aromatase	Inactive	0.98	Inactive	0.97
	Estrogen receptor alpha	Inactive	0.76	Inactive	0.88
	Estrogen receptor ligand binding domain	Inactive	0.94	Inactive	0.97
	Peroxisome proliferator activated receptor gamma	Inactive	0.96	Inactive	0.98
Tox21-Stress response pathways	Antioxidant responsive element	Inactive	0.99	Inactive	0.98
	Heat shock factor response element	Inactive	0.99	Inactive	0.98
	Mitochondrial membrane potential	Inactive	0.96	Inactive	0.93
	Phosphoprotein p53	Inactive	0.96	Inactive	0.92
	ATPase family AAA domain-containing protein 5	Inactive	0.99	Inactive	0.97
